# Barriers and facilitators to deprescribing in primary care: a systematic review

**DOI:** 10.3399/bjgpopen20X101096

**Published:** 2020-07-29

**Authors:** Alison Jayne Doherty, Paul Boland, Janet Reed, Andrew J Clegg, Anne-Marie Stephani, Nefyn Howard Williams, Beth Shaw, Lynn Hedgecoe, Ruaraidh Hill, Lauren Walker

**Affiliations:** 1 Faculty of Health & Wellbeing, University of Central Lancashire, Preston, UK; 2 University of Central Lancashire, Preston, UK; 3 Health Services Research, University of Liverpool, Liverpool, UK; 4 Oregon Health & Science University, Portland, Oregon, US; 5 University of Liverpool, Liverpool, UK

**Keywords:** primary care, review, deprescribing, polypharmacy, multimorbidity, primary health care, general practice

## Abstract

**Background:**

Managing polypharmacy is a challenge for healthcare systems globally. It is also a health inequality concern as it can expose some of the most vulnerable in society to unnecessary medications and adverse drug-related events. Care for most patients with multimorbidity and polypharmacy occurs in primary care. Safe deprescribing interventions can reduce exposure to inappropriate polypharmacy. However, these are not fully accepted or routinely implemented.

**Aim:**

To identify barriers and facilitators to safe deprescribing interventions for adults with multimorbidity and polypharmacy in primary care.

**Design & setting:**

A systematic review of studies published from 2000, examining safe deprescribing interventions for adults with multimorbidity and polypharmacy.

**Method:**

A search of electronic databases: MEDLINE, Embase, Cumulative Index of Nursing and Allied Health Literature (CINHAL), Cochrane, and Health Management Information Consortium (HMIC) from inception to 26 Feb 2019, using an agreed search strategy. This was supplemented by handsearching of relevant journals, and screening of reference lists and citations of included studies.

**Results:**

In total, 40 studies from 14 countries were identified. Cultural and organisational barriers included: a culture of diagnosing and prescribing; evidence-based guidance focused on single diseases; a lack of evidence-based guidance for the care of older people with multimorbidities; and a lack of shared communication, decision-making systems, tools, and resources. Interpersonal and individual-level barriers included: professional etiquette; fragmented care; prescribers’ and patients’ uncertainties; and gaps in tailored support. Facilitators included: prudent prescribing; greater availability and acceptability of non-pharmacological alternatives; resources; improved communication, collaboration, knowledge, and understanding; patient-centred care; and shared decision-making.

**Conclusion:**

A whole systems, patient-centred approach to safe deprescribing interventions is required, involving key decision-makers, healthcare professionals, patients, and carers.

## ​How this fits in

Previous reviews have explored patient and/or prescriber barriers and facilitators to safe deprescribing in primary care.^1–15^ However, to the authors’ knowledge, none have fully explored the barriers and facilitators to safe deprescribing interventions for patients with multimorbidity and polypharmacy at the different socioecological levels, and none have explored any health inequality issues involved. A comprehensive understanding of the barriers and facilitators (including health inequalities) at different socioecological levels is required to inform safe deprescribing intervention developments, implementation, and impact in primary care.

## Introduction

Polypharmacy, the use of multiple concurrent medications to manage multimorbidity, is a growing concern for healthcare systems globally.^16^ It affects some of the most vulnerable in society, often contributing to, and perpetuating, health inequality. Although older people are particularly affected by polypharmacy (with a prevalence of 30% to 60% in people aged ≥65 years in high-income countries),^14,17,18^ people with learning disabilities, younger people, and those from deprived communities may also have multimorbidity and have been prescribed polypharmacy.^16,19^ The growing impact of polypharmacy is widely recognised, reflecting ageing populations, the use of preventive medication, and fragmentation of care through condition-specific guidance.^20^ While polypharmacy may be appropriate in managing complex or multiple conditions, it presents risks and can be an inefficient use of resources.^20^ Inappropriate polypharmacy may increase treatment burden, undermine adherence to treatment, result in adverse drug-related events, and increase health service use.^21–24^ It is thought to contribute to around 8% of unplanned (Scottish) hospital admissions in older people on multiple medicines,^16^ and to the 47% increase in prescriptions dispensed in primary care (UK) over the decade from 2007 to 2017.^25^ Decreasing inappropriate polypharmacy has become a focus of national and international policy initiatives to improve health, reduce patient harm, and reduce healthcare costs.^24,26^

One way of reducing exposure to polypharmacy is through 'deprescribing'. Deprescribing involves the:

*'* *systematic process of identifying and discontinuing drugs when existing or potential harms outweigh existing or potential benefits within the context of an individual patient’s care goals, functional status, life expectancy, values, and preferences*.*'* ^27^

Care for patients with multimorbidity and polypharmacy predominantly takes place in primary care, with the GP undertaking a crucial role in managing patients’ medication.^8,28^ Despite the development of safe deprescribing interventions, uncertainty remains as to their effectiveness, and to the factors that may influence their implementation.^3,7,12^ This is particularly evident in primary care, where there is no consistent approach to deprescribing.^12^ This study systematically reviews the evidence on the barriers and facilitators (including health inequalities) to safe deprescribing interventions for adults (aged ≥18 years) with multimorbidity and polypharmacy in primary care.

## Method

This systematic review followed recognised guidance and reporting standards,^29,30^ with the methods described in a protocol registered on PROSPERO (reference number: CRD42019121848). Studies were identified through searches of five electronic databases, including MEDLINE (via Ovid), Embase, CINAHL, Cochrane, and HMIC (see Supplementary Appendix S1). Databases were searched from inception to 26 Feb 2019, and were limited to studies published in English. Other references were identified through handsearching specific journals (*BMJ Open* and *British Journal of Clinical Pharmacology*, between 2013 and 2019), and screening of reference lists and citations of included studies. All search results were stored in a Clarivate Analytics’ Endnote (version 9) database.

Studies were eligible if they were: studies of any design; included adults (aged ≥18 years) with multimorbidity and polypharmacy (≥2 long-term health conditions, and prescribed ≥4 of any type of medications), and/or their caregivers, and/or healthcare professionals who provide or deliver safe deprescribing interventions, such as GPs, nurses, and pharmacists; and included barriers and facilitators to safe deprescribing interventions, including medication reviews, decision support systems, pharmacist-led interventions, and screening tools. Settings included general practice-based settings, and GPs working in residential care settings for older people. Studies were excluded if they: involved patients in an exclusively end-of-life care setting; were in hospital-based settings; only assessed medication errors; were abstracts, editorials, commentaries, or opinion pieces; were published in a non-English language, or before 2000; or if the full text was not available.

Study selection occurred through two stages. First, titles and abstracts of papers from the searches were screened independently by two reviewers, using criteria specified prior to screening (see Supplementary Appendix S2). Second, full-text manuscripts of studies that met the criteria at the title and abstract screening stage were retrieved and screened independently by two reviewers using the same criteria. Data were extracted using a pre-piloted form by one reviewer, and checked by a second reviewer. Extracted data included: first author; year of publication; title; country of origin; journal; study aims and objectives, design, setting, participants, intervention, and intervention components; and barriers and facilitators reported.

Using an approach advocated by the Health Inequalities Assessment Toolkit,^31^ two researchers independently searched the included studies and extracted data for any reported evidence of health inequality barriers to safe deprescribing interventions.

Study quality was assessed independently by two reviewers using the Mixed Methods Appraisal Tool (MMAT) checklist. At each stage, discrepancies in decision-making were resolved through discussion, and where necessary, through recourse to a third reviewer. Study selection was managed using Covidence, with Microsoft Excel 2015 used to support the selection of papers, data extraction process, and the quality appraisal process.

Data analysis was undertaken using a thematic analysis approach.^32^ Two researchers independently developed themes. They compared themes and developed consensus through discussion. An additional reviewer was involved where necessary. NVivo (version 12) was used to support data analysis. Quotes were extracted from the included studies as supporting evidence for the themes identified.

The Socio Ecological Model (SEM) was used for the presentation of findings.^33^ SEM is a theory-based framework used for understanding the complexity of inter-related barriers and facilitators within the multiple levels of a whole system, such as a healthcare system.^34^ Logic models can illustrate the types of inputs and activities needed within the different SEM levels to move towards whole systems change and collectively agreed outcomes.^35^ The authors adapted a method from previous work in this field^36^ to build a logic model informed by the findings of this review.

## Results

**Figure 1.**
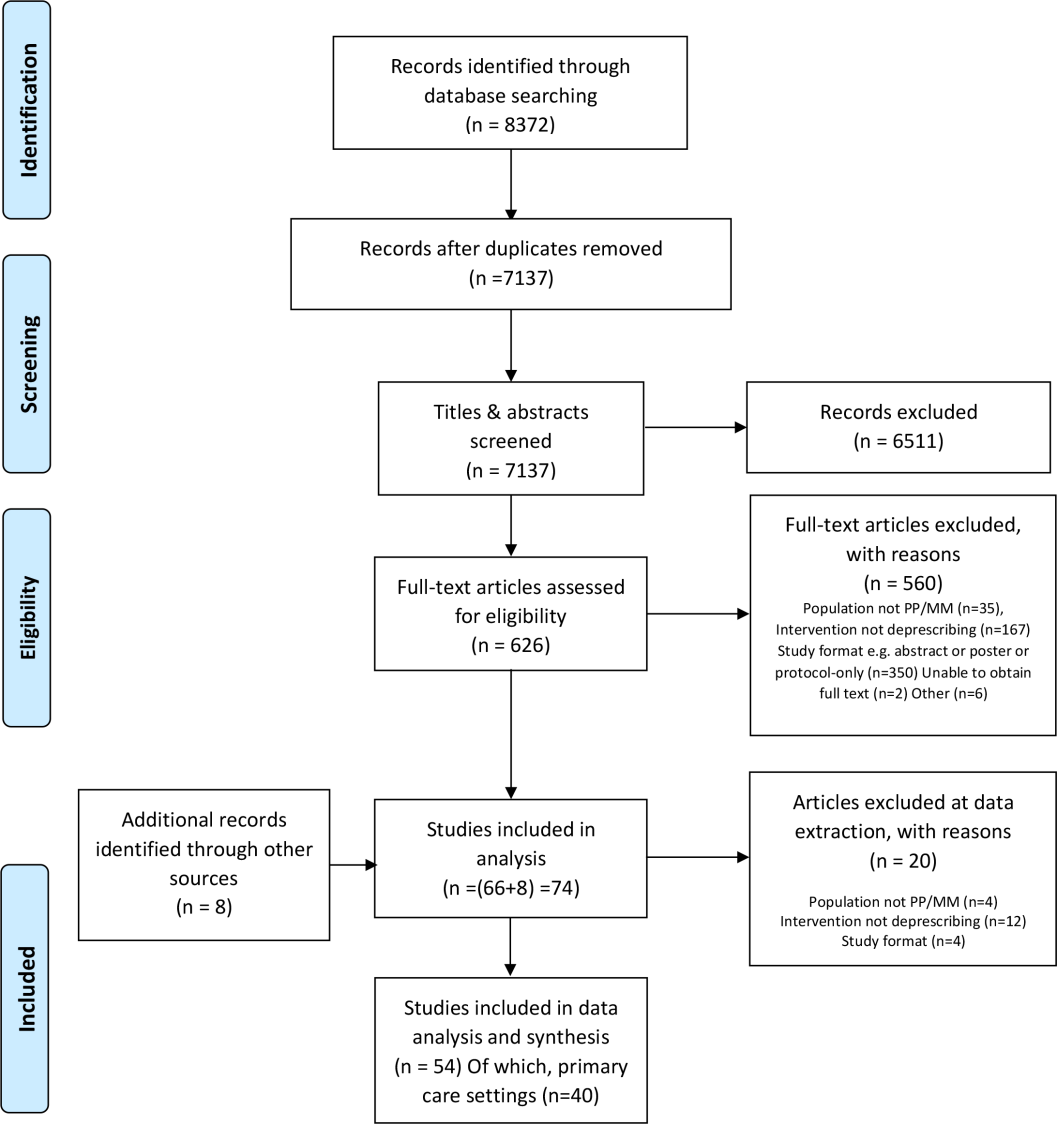
PRISMA flowchart

A combined total of 7137 papers were gathered from the database searches (after duplicate records removed). Screening titles and abstracts resulted in 626 papers for consideration of full texts. Other identification approaches, such as citation searching and reference list handsearching, identified a further eight papers for full text consideration. Of the total of 634 full text papers, 560 records were excluded. A total of 54 papers met the eligibility criteria for the review and reported sufficient information for quality appraisal and data extraction. Of these, 40 papers reported on barriers and facilitators to deprescribing in primary care. The review process is summarised in Figure 1. Supplementary Table S1 provides a summary of the 40 studies included in this review.

Data were heterogeneous and there were differences in data collection, data sources, and analysis across included studies. Of the 40 eligible studies, the outcomes from the MMAT quality appraisal process showed that 26 were rated as high quality (five stars), four were rated as good quality (four stars), six were rated as satisfactory (three stars), two were rated as poor (two stars), and two were rated as very poor (one star). Supplementary Table S1 includes the MMAT scores for these studies.

The studies countries of origin included: US (*n* = 7), Australia (*n* = 6), Germany (*n* = 5), the Netherlands (*n* = 4), Canada (*n* = 3), Republic of Ireland (n = 3), UK (*n* = 3), Belgium (*n* = 2), New Zealand (*n* = 2), Israel (*n* = 1), Norway (*n* = 1), Singapore (*n* = 1), Switzerland (*n* = 1), and Italy (*n* = 1). Publication dates ranged between 2010 and 2019. Most studies were published in 2017 (*n* = 9) and 2018 (*n* = 15). None were published between 2000 and 2010. Twenty-four studies used qualitative methods, including semi-structured face-to-face or telephone interviews (*n* = 18), and focus groups (*n* = 6); 12 studies used quantitative methods, including surveys (*n* = 10), one non-randomised controlled trial (RCT), and one RCT; and four studies used mixed methods, including RCT and survey (*n* = 1), and interviews and surveys (*n* = 3).

Interventions included medication reviews, screening tools, education interventions, and pharmacist integration interventions. The studies’ participants included GPs, patients, nurses, physicians’ assistants, pharmacists, and staff from long-term care facilities (or combinations of these participants). A total of 5516 participants were included: patients or their carers (*n* = 3673), GPs (*n* = 1208), nurses or physicians’ assistants (*n* = 351), pharmacists (*n* = 239), primary healthcare practitioners (no breakdown of professions provided*; n* = 26), and long-term care facilities’ staff (*n* = 19).

**Figure 2.**
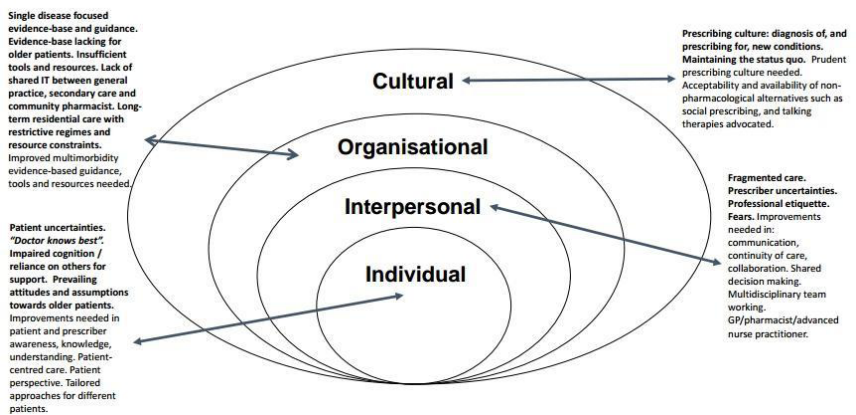
Socio-ecological approach to understanding barriers and facilitators to deprescribing in primary care for patients with multimorbidity and polypharmacy

The barriers and facilitators that emerged from the studies were complex and interlinked at different SEM levels. Fewer facilitators than barriers were described. Supplementary Table S2 presents a summary of data extracts from the included studies relating to each of the barrier and facilitator themes identified. Figure 2 provides a visual summary of these themes. The barrier and facilitator themes, including identified health inequalities, are discussed narratively below.

### Themes

#### Barriers

Several inter-related barrier themes existed at different cultural, organisational, interpersonal, and individual socioecological levels.

##### ​Cultural barriers

A prevailing culture encouraged the diagnosis of medical conditions and the prescribing of medications:^37,38^

*‘patients expected there to be* *”a pill for every ill*“ *and* ... *this expectation was exacerbated by direct-to-consumer advertising of medicines in New Zealand*.*’* ^39^

Studies described a trend towards the prescribing of medication for asymptomatic patients to prevent future morbidity and mortality,^40^ and a continuation of unnecessary preventive medicine in older patients.^41,42^

There was a lack of financial incentives for primary healthcare practitioners to address polypharmacy:

*’*… *there's no money involved in addressing polypharmacy … you could be completely cynical and turn round and go,* *“* *This patient gets 12 items, I get paid* *98* *p* *for each item I dispense. Why do I want to go out and tell this man he only needs seven of them?* *“’* (PCT2)^43^

Patients in some countries with free prescriptions (or discount cards for the costs of medications) were less likely to cease medications.^44^

##### ​Organisational barriers

Evidence-based guidance tended to focus on single disease management, which was difficult for GPs to apply in practice when patients often have more than one medical condition:

*‘Guidelines tend to be very disease-specific but how do you do something? How do you prescribe for somebody who has comorbidities and the guideline would seem to be talking against each other … where is the evidence base … ?’* (GP8)^43^

Health inequalities were identified for older people due to a lack of evidence-based guidance for older patients with multimorbidity and how best to deprescribe multiple medications in this population:^45–48^

*‘* *… the cardiology guidelines still recommend very restricted diets for patients with heart disease and those guidelines are appropriate for 50 year old overweight patients, but 85 year old patients with multiple chronic diseases are at risk of malnutrition so those guidelines are really not appropriate …* *’* (Clinical pharmacologist)^49^

Busy long-term residential care environments for older people with restrictive regimes, staffing shortages, high staff turnover, and problems accessing GP services also hindered safe deprescribing interventions for older patients with multimorbidity and polypharmacy.^50–52^

There was inaccurate information and insufficient communications technologies for primary healthcare practitioners to aid their deprescribing decision-making.^53^ Other resource constraints included limited GP time and a lack of non-pharmacological alternatives, such as talking therapies for mental health.^43,47,54,55^

##### ​Interpersonal barriers

Studies highlighted a fragmentation of care due to a lack of joined-up communication and collaboration between different prescribers in different healthcare settings.^39^ With multimorbidity, several healthcare providers were involved in a patient’s treatment and communication across different healthcare settings was often poor.^42,45,55,56^ In some cases, it was the patient who provided the GP with information due to the lack of up-to-date communication between the GP and other healthcare professionals involved in their care.^49^

Some primary healthcare practitioners struggled to find the right 'language' to initiate deprescribing discussions with patients.^54^ Uncertainties and a lack of knowledge, awareness, guidance, and tools and resources for deprescribing made it easier for the GP to continue to prescribe and to maintain the 'status quo'.^39,40,54,57–59^ Uncertainties also affected pharmacists’ confidence in making recommendations to GPs.^60^

Opportunities for deprescribing were sometimes lost due to GPs’ lack of direct contact with patients who receive repeat prescriptions.^57,61^

GPs were reluctant to stop a medication started by another specialist in a different healthcare setting:^41,51,56^

*‘GPs outlined challenges in terms of professional boundaries with hospital prescribers and some GPs were reportedly unwilling to challenge recommendations from secondary care* ... *’* ^43^

Uncertainties gave rise to GPs’ fears over adverse effects from stopping medications, legal repercussions, and of being perceived by their patients as disengaging or not caring about them if they broached the subject of stopping medications.^41,42,47,50,56,62^

##### ​Individual barriers

Patients may not have been able to state the reasons why they were on certain medications or may not have known or cared about their side effects, and this hindered deprescribing.^61,63,64^ Some primary healthcare practitioners and patients themselves felt that patients were disinclined to cease medications, particularly those they had been taking over many years, and they may have also taken 'over-the-counter' medications unbeknown to the GP:^38,56^

*‘* *Concerning diclofenac for the older patients it simply is like that, he* [the patient] *just doesn’t want* [to discontinue the drug] *and says,* *“* *you can’t take this away from me.* [I am] *free of pain for the first time in* *7* *years* *. I need that.* *”*’ (GP 10)^57^

Primary healthcare practitioners perceived that patients had just followed 'doctor’s orders' without questioning decisions, or had not taken (or were unable to take) an active part in decision-making.^53,59^ However, GPs did not always ask for their patients’ views.^57^ Younger adults were more likely to have an active role in their medication management compared to older people,^56,65^ and older age combined with lower levels of education was found to be a significant barrier to safe deprescribing interventions.^42^

Health inequality barriers to safe deprescribing were identified for patients from migrant communities,^56^ for patients with mental health issues,^55^ and for patients with sensory impairments and/or cognitive impairments.^55,66^

Several studies commented on primary healthcare practitioners’ attitudes and assumptions towards older patients, for example, having the assumption that medication side effects were less important in older patients.^42,45,59,61,65^ GPs were often reliant on the carers of older patients,^65^ but carers may have lacked involvement in medication management due to issues such as time constraints.^47,56^ GPs’ deprescribing recommendations for people with impaired cognition may also be hindered by opposition from their families or carers:^40,42,54,59,67^

*‘I stopped metformin in a 90-year-old with dementia, daughter complained, made me wary to deprescribe* *.’* (GP)^67^

#### Facilitators

The facilitators to deprescribing in primary care identified by this review provide suggestions for action at different socioecological levels.

##### ​Cultural facilitators

Studies found that a change in culture was required. The prevailing culture of diagnosis and prescribing, and the associated attitude of 'more is better', needed replacing with a more prudent prescribing culture:

*‘… activating patients to become more involved in medicines management and alert to the possibility that less might be better.* *’* ^39^

##### ​Organisational facilitators

Better evidence-based guidance on multimorbidity was required, including advice on common drug-drug interactions, and when and how to start and stop different medications.^39,68^ Guidance should be in a format that is easily accessible during discussions between prescribers and patients:

*’Deprescribing should like be a component of all treatment guidelines … I feel focusing in on a few drugs, developing the methods for deprescribing guidelines and getting a few guidelines into play is what is needed to catalyse a larger deprescribing movement* ... *I really think all prevention-oriented meds deserve a deprescribing guide*.*’* ^48^

Stopping medications was easier for patients who prioritised 'reducing other symptoms' as their most important health outcome.^46^

Guidance must address the practical skills and tools needed by GPs and other primary healthcare practitioners, as well as improving their knowledge base.^39,56^ Studies identified a need for a medication-specific tool to support shared decision-making in primary care.^39,58,69^ Better continuity between primary care and other healthcare settings, and protected time were needed.^41,62^ Studies also highlighted the need for access to alternative non-pharmacological options.^39,40,48^

##### ​Interpersonal facilitators

Improved communication between primary healthcare practitioners and their patients was a key facilitator to deprescribing.^44,63,65^ Patients generally trusted GPs and were open to discussions about deprescribing, but GPs needed appropriate tools to help them start such conversations.^47^ Patients also needed information to be involved in such conversations, but some patients had not received sufficient information about the effects of their medications from either their GP or pharmacist.^59^

Tipping points, such as deterioration in an existing multimorbidity, were found to be useful in initiating discussions about deprescribing between GPs and their patients.^70^

Initiation of stepwise reductions in certain medications by GPs was useful too:

*‘This suggests that a stepwise reduction of medication was followed and may be a good approach to stop symptom-relieving medication.* *’* ^46^

Continuity of care was important. This should not just be between the GP and patient, but also between the GP and pharmacist, and the GP and other specialists involved in treatment.^65,71,72^ However, continuity of care was challenging due to fragmented care, professional boundaries, limited time, and other resource constraints.

Discussions with other experienced colleagues,^54^ wider practice-based discussions,^45^ and close collaboration with community pharmacists (CPs) and other interdisciplinary services facilitated deprescribing decisions:^40,41,52,73^

*‘Several clinicians said they beneﬁted from having on-site pharmacists review patients* *’* *medications lists and provide guidance on tapering regimens or drug interactions. Other interdisciplinary services that were perceived as helpful included: on-site continence nurses or physiotherapists … and social workers to help caregivers access resources or understand what to expect as dementia progresses.’* ^41^

CPs could make recommendations to GPs to cease potentially inappropriate medications.^70^ They had more time for medication reviews but lacked GPs’ detailed knowledge of individual patients. To be successful the CP needed access to the practices’ electronic health records,^73^ but this is not current routine practice.

Time for interdisciplinary case conferences was recommended.^52^

##### ​Individual facilitators

GPs needed improved information and guidance on deprescribing (including risks and benefits) so that they were better informed and more confident in their own deprescribing actions.^37,57^ Seeking guidance from experienced peers and other colleagues was helpful and improved GPs’ self-efficacy.^45^ Improvements in how information is conveyed could help manage carers’ expectations for those with relatives in long-term care.^51^

GPs, nurses, and pharmacists indicated that medication reviews could be prioritised for mental health, cardiovascular, gastroenterological, and neurological conditions.^48^

Patient involvement in decision-making and patient-centred care was considered essential by GPs:

*‘This study showed that GPs consider* *“patient-centredness* *“* *as the most important goal when taking care of patients with multimorbidity.’* ^55^

Trust in the GP influenced a patient’s willingness to stop medications. Patients who were informed and involved in decision-making valued the extra attention given by their GP and were more likely to act on their GP’s recommendations.^41,53,54,72^ Prescribers demonstrated an awareness of the need for individually tailored prescribing as a valued part of patient-centred practice:

*‘The* [different] *participant types identified in this study suggest that deprescribing should be tailored to older adults* *’* *understanding of their medicines, their attitudes towards medicines and deprescribing, and their preferred participation in decision-making.’* ^59^

*’Future work is needed to improve provider abilities to elicit a patient’s desired level of involvement in making decisions to continue or discontinue medications.’* ^53^

Many patients wanted to take fewer medications:

*‘* *Cause it’s a pain to take these pills. I mean I have to take them, and it’s a habit right now, but if I didn’t have to take them, I wouldn’t miss them* *.* *’* (V10)^53^

*‘... 72.8* *%* *still had the desire to reduce the number of medications they were taking. A quarter of participants (25.0%) felt that they might be taking one or more medications that they no longer needed, and* *30* *%* *felt that one or more of their medications were giving them side effects.’* ^44^

Some patients wanted an active role in the decision-making process, but they needed to understand more about the benefits and risks of stopping their medications:

*‘* *Well, they would have to show me where it’s beneﬁtting me to stop. I mean I just wouldn’t walk out. I would say* *,* *“Wait a minute, why do you say this. Tell me why this here is better than this one, or I don’t need this.”* *’* (V4)^53^

Patients who have an ability to question their GP about their medication, and who take an active role in decision-making, can facilitate safe deprescribing interventions, and reinforce and benefit the doctor-patient relationship.^48,65^

## Discussion

### Summary

This review found a complex of barriers and facilitators to safe deprescribing interventions for people with multimorbidity and polypharmacy. Health inequality barriers identified related to issues for older people including, for example, a lack of evidence-based guidance for older people with polypharmacy and multimorbidity; certain attitudes and assumptions among some healthcare professionals towards older people; challenges with care continuity support for older people; and barriers for those with cognitive and/or sensory impairments (mainly older people, but not exclusively). There was also evidence of health inequalities experienced by patients with multimorbidity, polypharmacy and mental health conditions, and patients from migrant communities with multimorbidity and polypharmacy.

**Figure 3.**
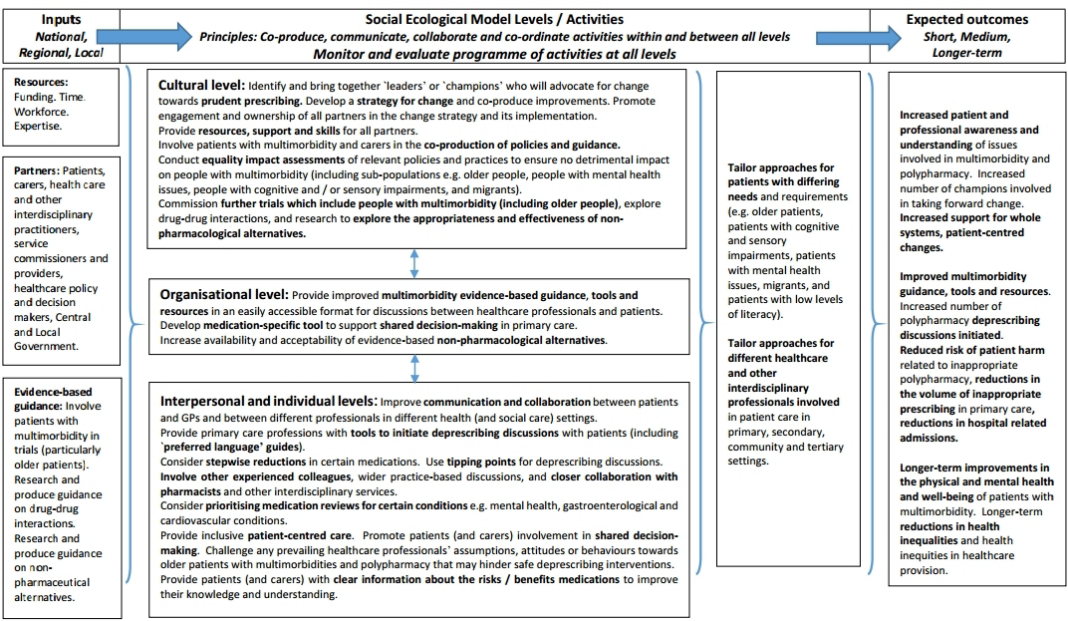
Logic model

The complexity of the SEM level barriers, including health inequalities, identified by this review indicates the need for a whole systems approach. This approach explores barriers within a whole system, assesses the inter-relationships between these barriers, and considers ways of achieving desired change and outcomes.^33,74^ Figure 3 presents a logic model that has been informed by this review’s findings. The model suggests the types of inputs, activities at different SEM levels, and interconnectivities needed to bring about whole systems change and desired outcomes for safe deprescribing in primary care. The model shows the types of inputs (for example, funding, evidence-base, key stakeholders) needed to overcome identified barriers and effect change. Using these inputs, those involved may engage in a range of activities at different SEM levels to achieve expected outcomes. This model recognises the varied contributions at different SEM levels and the contributions’ interconnectivities, and provides a 'route map' for mobilising action and moving forward. If effectively co-produced, communicated, coordinated, and collaboratively undertaken and owned, this whole systems approach may contribute towards improvements in safe deprescribing for people with multimorbidity and polypharmacy in primary care.

### Strengths and limitations

To the authors’ knowledge, this is the first systematic review incorporating a range of study types to synthesise the different SEM barriers and facilitators to safe deprescribing interventions for patients with multimorbidity and polypharmacy in primary care. It may also be the first systematic review to explore the health inequalities that can affect patient care.^75^ It provides a timely summary of the recent literature from 2000–2019. It includes the different perspectives of primary healthcare professionals, prescribers, patients, and carers. However, the search was limited to studies published in English. There may be other relevant studies published in other languages. Studies identified were all from high-income countries, and are therefore not representative of all international contexts. More global studies are needed. Many of the included studies used qualitative methods, such as questionnaires, to explore participants’ views, but implementation studies are needed to explore what happens in practice.

### Comparison with existing literature

Previous reviews have made similar deductions, including the paucity of evidence-based guidelines for deprescribing,^2,11,28^ concerns over the lack of guidance on the interactions between different medications,^11^ professional etiquette barriers, and limited non-pharmacological options.^2,15^ Previous reviews have found that GPs receive poor communication from other healthcare providers about patients with multimorbidity.^1,11^ Other reviews have identified the need for integrated patient care across services, enhanced time-saving information technology, and other tools and resources to support communication and collaboration between different providers.^7,11^ The organisation of healthcare systems has been described as poorly suited to deprescribing.^13^ Previous reviews have similarly highlighted the importance of good communication between the GP and their patient,^76^ and the need for modified, tailored advice for patients with multimorbidity.^1,11^ Uniquely, this review collated evidence on barriers and facilitators to safe deprescribing interventions in primary care from different SEM levels, and from the different perspectives of healthcare professionals, prescribers, patients, and carers in primary care. It explored, and found, evidence of health inequality issues for groups of people with multimorbidity and polypharmacy, including older people, people with sensory and/or cognitive impairments, people with mental health issues, and migrants. The review found that a whole-systems, patient-centred approach is required to address the complexity of identified systems-level barriers to safe deprescribing interventions in primary care.

### Implications for research and practice

With the right support and engagement, deprescribing should become part of a wider movement towards more sustainable lifestyles, with a focus on reducing patient harm and the detrimental impact on healthcare resources. This requires involvement of those with the ability to change culture and practice. Specific considerations are:

- While key policies in the UK advocate shared decision-making, such policies require resourcing and attitudinal shifts at all levels to become part of routine practice.^77^
- The UK’s incentive schemes for GPs have rewarded general medical practices for quality care provision, but primarily focus on single-disease management, for example, cardiovascular disease management utilising therapeutic interventions.^78^ However, GPs could be rewarded for rationalising inappropriate polypharmacy, and a broader view is needed on what constitutes 'quality care' in medication management, which must include patient-centred measures.^78^
- More clinical trials involving patients with multimorbidity are needed (including older people with multimorbidity and trials exploring drug-drug interactions) to inform evidence-based guidance. There is emerging evidence that this evidence-gap has started to be addressed: 38 studies containing the term 'deprescribing' were listed on the recognised clinical trials research registry (https://clinicaltrials.gov; searched December 2019). Seven of these specifically target patients with multimorbidity. There is also, for example, a collaborative project specifically targeting patients with dementia and multimorbidity (clinicaltrials.gov identifier: NCT03984396).
- Further co-development and dissemination of safe deprescribing interventions, shared decision-making tools, and resources is required in primary care.^79^
